# Neighborhood Characteristics: Influences on Pain and Physical Function in Youth at Risk for Chronic Pain

**DOI:** 10.3390/children3040035

**Published:** 2016-11-19

**Authors:** Cathleen Schild, Emily A. Reed, Tessa Hingston, Catlin H. Dennis, Anna C. Wilson

**Affiliations:** 1 Pacific University, Department of Clinical Psychology, Forest Grove, OR 97116, USA; schi6727@pacificu.edu; 2 Oregon State University, Department of Psychological Science, Corvallis, OR 97331, USA; emilyreed9394@gmail.com; 3 George Fox University, Department of Psychology, Newberg, OR 97132, USA; hingstot@ohsu.edu; 4 Oregon Health & Science University, Institute on Development and Disability, Department of Pediatrics, Portland, OR 97239, USA; dennica@ohsu.edu

**Keywords:** walkability, attitudes about physical activity, chronic pain, adolescents

## Abstract

Neighborhood features such as community socioeconomic status, recreational facilities, and parks have been correlated to the health outcomes of the residents living within those neighborhoods, especially with regard to health-related quality of life, body mass index, and physical activity. The interplay between one’s built environment and one’s perceptions may affect physical health, well-being, and pain experiences. In the current study, neighborhood characteristics and attitudes about physical activity were examined in a high-risk (youths with a parent with chronic pain) and low-risk (youths without a parent with chronic pain) adolescent sample. There were significant differences in neighborhood characteristics between the high-risk (*n* = 62) and low-risk (*n* = 77) samples (ages 11–15), with low-risk participants living in residences with more walkability, closer proximity to parks, and higher proportion of neighborhood residents having college degrees. Results indicate that neighborhood features (e.g., walkability and proximity to parks), as well as positive attitudes about physical activity were correlated with lower levels of pain and pain-related disability, and higher performance in physical functioning tests. These findings suggest that the built environment may contribute to pain outcomes in youth, above and beyond the influence of family history of pain.

## 1. Introduction

Neighborhood characteristics have an impact on the health outcomes of residents, in terms of health-related quality of life (HRQOL), body mass index (BMI) and the experience of chronic pain [1,2,3]. The accessibility of neighborhood amenities (e.g., being able to walk to the grocery store) is thought to be related to physical activity level among residents, leading to changes in health outcomes. To quantify the accessibility of neighborhood amenities, such as schools, grocery stores, and restaurants, walkability scores can be assigned to each physical address based on ease of access without a car.

Adolescents may be especially vulnerable to the effects of a neighborhood with low walkability [4]. Low walkability indicates that places of interest, including parks, schools, and stores, are not easily accessed on foot. Prior to receiving a driver’s license or having other autonomous transportation options, young adults have limited access to amenities outside their neighborhood. By living in a walkable neighborhood, adolescents have more autonomy compared to those living in a less walkable neighborhood. Therefore, the accessibility of the neighborhood in which adolescents live is vital for promoting and providing outlets for adolescent physical activity.

Research exploring socioeconomic status (SES), with respect to both family and the neighborhood, has found that SES has a large effect on the resources available to lead healthy lives. Neighborhoods with a higher median SES are associated with a higher density of recreational facilities, parks and walkable pathways, and are believed to promote healthy lifestyles more than neighborhoods with a lower median SES [5]. Families with higher SES are better able to afford living in neighborhoods where there is a more developed built environment (e.g., developed sidewalks and landscaping) and more amenities. Neighborhoods that are associated with a lower socioeconomic status feature a less developed built environment (e.g., undeveloped sidewalks and fewer trees) [6], which can lead to lower activity rates, higher rates of disability and higher BMI trends over time [2]. Children and adolescents raised in underdeveloped neighborhoods are more likely to be inactive or to be overweight than those living in highly developed neighborhoods [7].

Additionally, there is a direct relationship between the experience of chronic illness and SES in the United States; people of lower SES experience more chronic illness than their more affluent counterparts [8,9,10]. In particular, chronic pain conditions are experienced disproportionately by people in the lowest income bracket (i.e., people that are unemployed and receiving disability) [11,12]. Therefore, because SES is related to both neighborhood amenities and to conditions such as chronic pain, it is necessary to explore the associations between the neighborhoods people live in and their risk for experiencing chronic pain.

Previously held beliefs about physical activity have also been shown to influence the activity level of adolescents. Positive attitudes about physical activity, as well as the intention to perform physical activity, were found to be consistent indicators of physical activity among adolescents [13]. The perception of having available physical activity resources is nearly as important as the resources themselves [5]. Additionally, when a neighborhood features a variety of physical activity amenities, such as parks or sport facilities, adult and adolescent residents are more likely to meet physical activity guidelines [4,5]. Having access to at least one recreational facility within walking distance significantly decreases the odds of being overweight, compared to a similar population with no access to recreational facilities [14]. Overweight and obese weight status has been associated with increased risk for a variety of chronic pain conditions, as well as increased pain and pain-related disability [15,16,17,18].

Chronic pain conditions have been found to negatively impact a child’s physical activity level. Children with chronic pain exhibit a lower peak activity level, and are sedentary for more minutes a day than their healthy counterparts [19]. Lower activity levels have been associated with more perceived physical limitations among participants [19], indicating a connection between physical activity and perceived barriers to activity. Limited research has examined neighborhood level contributions to pain and related outcomes in pediatric chronic pain samples. Among youth with sickle cell disease, living in a distressed neighborhood was found to be associated with diminished physical HRQOL and increased reports of pain [1]. Thus, neighborhood level characteristics may be associated with chronic pain and related disability, particularly in high-risk samples, or in individuals who are at increased risk for pain due to the presence of comorbidities or other risk factors (e.g., low physical activity, or high BMI).

The current study has three primary aims. The first is to compare neighborhood characteristics between two parent-child samples: those in which the parent had a chronic pain condition (high-risk group) and those not impacted by parental chronic pain (low-risk group). It is well-established that youth who have a parent with pain are at increased risk for developing pain problems [20]. Differences in neighborhood characteristics associated with parental pain may confer additional risk for pain, disability, and poor physical function outcomes. It was hypothesized that high-risk youth would live in neighborhoods with lower walkability and more restricted access to recreation compared to low-risk youth. The second aim is to examine whether neighborhood level sociodemographic factors (median income, population education) contribute to child pain and disability above and beyond family socioeconomic factors (i.e., family income and parental education). The third aim is to examine the contribution of neighborhood built environment (e.g., walkability and access to recreation) and youth’s attitudes about physical activity to youth pain, activity limitations, and physical function outcomes. Physical function outcomes included laboratory-based tests of physical function, and self-report of physical activity. It was hypothesized that both neighborhood-level factors and individual attitudes would contribute to outcomes, above and beyond risk group membership and BMI.

## 2. Materials and Methods

### 2.1. Participants

The data used was taken from a larger prospective study examining the impact of parental chronic pain on youth. Data from the baseline time point of the larger study was used in the current analysis. Children of parents without a chronic pain condition—the low-risk study group—was made up of parent–child dyads (*n* = 77) and children of parents with a chronic pain condition—the high-risk study group—also consisted of parent–child dyads (*n* = 62). All study procedures were approved by the institutional review board (No. IRB#00005973) at Oregon Health & Science University in Portland, Oregon.

All participants were recruited from a major metropolitan area in the Pacific Northwest of the United States. Flyers explaining the study purpose were posted in clinics that specialized in multidisciplinary chronic pain services. Potentially eligible participants were identified through the clinic medical records and then sent a flyer that advertised the current study. Healthy parents and guardians were recruited through study flyers posted in the community and the university’s research website.

Inclusion criteria specified that participating parents in both groups had to have a biological child between the ages of 11 and 15 who was willing to participate. Inclusion in the chronic pain group required that parents must have had pain for a period of three months or longer, and had to be receiving specialty medical care for their pain. Parents in the healthy group were excluded if they reported historical or current chronic pain that persisted for a period of three months or longer. Parents in the healthy group were also deemed ineligible if they stated that the child’s other parent (biological or a co-habiting parent, such as a step-parent) had a history of chronic pain. Additional exclusionary criteria for both groups included the presence of chronic or serious illness (e.g., cancer, or arthritis), non-fluency in English, or cognitive impairment of either the parent or child.

### 2.2. Procedure

Potential participants called the study phone number or provided their contact information on the study website. A telephone interview was conducted with potential participants to evaluate both inclusion and exclusion criteria, verbal consent was attained, and a study visit was scheduled for data collection. Of the families who completed phone screens, three failed the screen because the child in the target age range was not a biological child (adopted or other biological relation). Two families were excluded from the healthy sample due to a biological parent having a history of chronic pain. During the phone screens, no participants declined participation; however, three families were not enrolled post-phone screen due to scheduling conflicts and illness. During the study visit written consent was attained from parents and assent was attained from child participants. Parents and children completed pain and physical activity questionnaires via REDCap (Research Electronic Data Capture), a secure web-based computerized survey system used for collecting and managing study data [21]. Children participated in standardized tests of physical function and had their height and weight taken during the study visit. Parent and child participants were compensated for participation in the form of a gift card to a local store.

### 2.3. Measures

#### 2.3.1. Sociodemographic Characteristics

Parent participants reported on individual items assessing sociodemographic characteristics, including parent and child ethnicity and racial background, parent education level, parent marital status, and family income. Parents also reported on their child’s sex and date of birth, and on their own sex and date of birth, as well as residential address. Birth dates were used to calculate ages of participants on the date of visit.

#### 2.3.2. Pain Characteristics

Adolescents were asked to describe pain characteristics of the last three months using a pain questionnaire. Children reported on pain location(s), frequency, and usual pain intensity. Pain location was marked on a validated body outline and pain location followed the nine standardized body regions identified by Lester and colleagues [22]. Pain frequency items are based on questionnaire items used in the World Health Organization’s survey on health behaviors in school-age children [23], which have been used to assess pain frequency in this age group [24]. Adolescents were asked to report pain frequency in the past three months with close-ended response options ranging from 0 (never) to 6 (daily). Adolescents were asked to report how much pain they usually have from aches and pains; usual pain intensity was reported using a 10-point Numeric Rating Scale (NRS; 0–10), ranging from 0 (No Pain) to 10 (Worst Pain Possible) [25,26]. 

#### 2.3.3. Pain-Related Activity Limitations

Adolescents reported on pain-related activity limitations using the Children’s Activity Limitation Interview (CALI-21), a 21-item inventory of perceived difficulty with activities of daily living because of pain. Each activity was rated on a 5-point scale (0–4), where 0 represented no difficulty and 4 represented extreme difficulty. Higher scores indicate a greater perceived impairment. The CALI-21 has excellent internal consistency, and moderate test–retest and cross–informant reliability [27,28].

#### 2.3.4. Self-Report of Physical Activity

Adolescent self-reports of physical activity were assessed using the International Physical Activity Questionnaire (IPAQ-7). Adolescents reported on physical activity undertaken across various domains including moderate and vigorous exercise, walking and sitting, and transport-related activity in the last seven days. The IPAQ-7 provides time spent in physical activity of different intensities. This time is then multiplied by multiples of the resting metabolic rate (METS), which measure typical energy expenditure at the various physical activity intensity levels. This 7-item questionnaire has adequate reliability and has been validated with objective measures of energy expenditure in adolescents [29,30]. The total MET-minutes per week score was used in the current analyses.

#### 2.3.5. Attitudes about Physical Activity

Adolescent self-reports of attitudes about physical activity were assessed using the Attitudes about Physical Activity-Child Report (APA-C). The APA-C is a 14-item questionnaire that assesses negative and positive attitudes toward activity. Each item was rated on a 5-point scale (1–5), where 1 represented disagree a lot and 5 represented agree a lot. This questionnaire has good reliability and validity [31].

#### 2.3.6. Body Mass Index (BMI)

Child height (centimeters) and weight (kilograms) were taken by trained research assistants at the study visit. BMI percentile for exact age was calculated using height, weight, date of birth and date of measurement via the Center for Disease Control’s online pediatric BMI calculator [32]. BMI percentile was used in the current study.

#### 2.3.7. Tests of Physical Function

Trained research assistants administered tests of physical function. A standardized, scripted set of instructions was given to each participant. Adolescents were asked to complete a sit-to-stand test that requires sitting in a chair with feet on the ground and knees at approximately a 90 degree angle. From a seated position, participants stood up and sat back down as quickly as possible for a 1-minute period. A trained research assistant recorded how many times each participant completed a full sit-to-stand motion. The sit-to-stand task is a commonly used test of physical function and is associated with exercise capacity, functional balance, and physical health-related quality of life [33,34,35].

Each participant also completed a timed 10-meter walk. They were instructed to walk a distance of 10 meters as quickly as possible. A trained research assistant timed how long it took for the walk to be completed in minutes and seconds. Timed fast walk performance tests are used frequently for assessing walk speed and other gait characteristics in children and adults, and norms based on age are available [36,37,38].

#### 2.3.8. Neighborhood Characteristics

##### Walkability

Individual residential street addresses were used to obtain a Walk Score (Walk Score, Seattle, WA, USA) for each participating dyad. Walk Score is a patented methodology that uses data from a variety of sources, including online map systems, USA Census data, and information from educational databases to analyze walking routes to a variety of amenities from a given street address. Scores are assigned into five categories based on their position in a hierarchy, ranging from very car dependent (scores from 0 to 24) to walker’s paradise (scores from 90 to 100) on a continuous 0–100 scale. In the lower-scored categories, transportation is needed to complete most or all errands outside the home. In the higher-scored categories, most errand locations (e.g., stores, schools, or recreation facilities) are within walking distance. Increasing Walk Score indicates that the corresponding location is closer to amenities [39]. This methodology has been validated in a number of metropolitan areas [40]. The continuous Walk Score was used in the current analyses.

##### Proximity to Parks

The Walk Score program also provides an exact distance (in miles) from each participant’s street address to the nearest park or public greenspace. For the purposes of this study, this distance was coded into a Park Score ranging from 0 to 2, with higher scores indicating closer proximity to parks, as follows: an address with a park within 0.5 miles was assigned a Park Score of 2, an address with a park between 0.5 miles and 1 mile away was assigned a Park Score of 1, and if the nearest park or recreational facility was located more than 1 mile away from a participant’s address it was assigned a Park Score of 0.

##### Census Neighborhood Data

The United States Census database (i.e., Census-Explorer, maintained by the USA Census Bureau, USA Department of Commerce, Economics & Statistics Administration, Suitland, MD, USA) was used to obtain demographic information about participants’ neighborhoods. Using Census-Explorer, participant addresses were entered and neighborhood level demographic information (percent of residents below poverty level, median income, percent college graduates) was extracted. This census tract-level information was obtained for each participant’s primary residential address to characterize the sociodemographic characteristics of the adolescent’s neighborhood.

### 2.4. Analyses

All analyses were conducted using IBM SPSS Statistics version 22.0 (IBM Corporation, Armonk, NY, USA). All data were screened for normality, appropriate ranges, and univariate outliers prior to performing analyses. Descriptive statistics were performed to characterize the sample. Independent samples *t*-tests were performed to examine potential group differences in neighborhood characteristics between the high-risk and low-risk groups. Multiple linear regressions were used to test whether median income and percent college graduates (neighborhood level sociodemographic factors) contribute to child pain and disability, above and beyond family income and parental education (family socioeconomic factors) and risk group. Multiple linear regressions were then performed to examine the contribution of neighborhood built environment (e.g., park proximity and Walk Score) and youth’s positive and negative attitudes about physical activity to pain, disability, and physical function outcomes, after controlling for risk group and BMI. Physical function outcomes included lab-based tests of physical function and self-report of physical activity.

## 3. Results

### 3.1. Descriptive Statistics

The sample consisted of adolescents from the high-risk group (*n* = 77) and low-risk group (*n* = 62). Mean age of the adolescent participants was 13.44 years (Standard deviation (SD) = 1.47), and 59.7% of the sample was female. The majority of participating adolescents reported Caucasian racial background (82.7%), with others reporting mixed race (10.8%), African American (3.6%), Asian (2.1%), and American Indian/Alaska Native (0.7%) racial background. A minority of the sample reported Hispanic ethnicity (7.2%). The high-risk and low-risk groups did not differ from each other on age, sex, ethnicity, or race.

### 3.2. Neighborhood Characteristics between High-Risk and Low-Risk Adolescents

Independent sample *t*-tests were used to compare the high-risk group to the low-risk group on the following neighborhood characteristics: Walk Score, percent below poverty level, median household income, and percent college graduates (see Table 1). There was a difference in the park scores between the groups, with the low-risk group having a significantly higher park score; *t*(134) = 2.43, *p* = 0.016. Additionally, there was a difference in the Walk Score for the groups, with the low-risk group having significantly higher scores; *t*(134) = 3.70, *p* < 0.001. There was also a significant difference found in the neighborhood percentage of college degrees, with the high-risk group having a lower percentage of college degrees than the low-risk group; *t*(135) = 2.51, *p* = 0.013. The percentage of the neighborhood population in poverty and neighborhood median income was not significantly different between study groups.

### 3.3. Contribution of Neighborhood Sociodemographic Factors to Child Pain and Disability

A multiple regression analysis was performed to evaluate the extent to which neighborhood level sociodemographic factors (i.e., median income, or percent college graduates) contributed to child pain and disability above and beyond family level sociodemographic factors (e.g., family income and parental education). Contrary to hypotheses, the analyses showed that after accounting for the significant effect of risk group, no family or neighborhood level income or education variables contributed significantly to adolescent pain intensity, pain frequency, or pain-related activity limitations. The study risk group was a significant predictor of pain intensity (β = 0.28, *p* = 0.006), pain frequency (β = 0.29, *p* = 0.003), and pain-related activity limitations (β = 0.40, *p* < 0.001), with high-risk group status being associated with increased pain and activity limitations.

### 3.4. Contribution of Walkability, Distance to Parks, and Attitudes about Physical Activity

Models of these adolescent outcomes included the same three steps. The first step included study risk group and BMI percentile, the second step included neighborhood walkability and proximity to parks, and the third step included adolescent positive and negative attitudes about physical activity. In a multiple regression model predicting adolescent’s usual pain intensity, negative attitudes about physical activity contributed significantly to higher usual pain intensity scores after controlling for study group, which also made a significant contribution. Overall, this model accounted for 20.2% of the variance in pain intensity scores (see Table 2). In a model predicting adolescent’s usual pain frequency, study group contributed significantly with the high-risk group having more frequent pain at entry in the model. Proximity to parks was also associated with pain frequency, such that closer proximity to parks (as assessed by park score) was related to lower pain frequency scores. In the final step of the model, higher negative attitudes about physical activity were significantly associated with increased pain frequency. Overall, this model accounted for 31.3% of the variance in pain frequency (see Table 3).

In a model predicting pain-related activity limitations, study group made a significant contribution with the high risk group reporting higher activity limitations. After controlling for group status and BMI percentile, proximity to parks also contributed significantly to activity limitations. Closer proximity to parks was associated with decreased activity limitations. Negative attitudes about physical activity also made a significant contribution to activity limitations in the final step of the model, such that increasing negative attitudes were associated with increasing activity limitations. Overall, this model accounted for 39.9% of the variance in activity limitations scores (see Table 4).

Results from a model predicting adolescents’ sit-to-stand performance scores showed that study group was associated with performance in the first two steps of the model, such that the high risk group completed fewer sit-to-stand motions during the task. BMI percentile was also associated with task performance such that youth with higher BMI percentile completed fewer sit-to-stand movements. Neighborhood walkability and proximity to parks did not contribute to performance on this task. However, in the final step of the model, negative attitudes about physical activity were associated with performance such that more negative attitudes were related to poorer sit-to-stand task performance. Overall, this model accounted for 21.5% of the variance in sit-to-stand performance (see Table 5).

In a model predicting timed 10 m walk performance, the high-risk group demonstrated significantly slower walk times. Additionally, neighborhood walkability contributed to walk times such that higher neighborhood walkability was associated with better performance in this task. In the final step of the model, positive attitudes about physical activity were significantly associated with shorter walk times, while negative attitudes about physical activity were significantly associated with longer walk times. Overall, this model accounted for 18.0% of the variance in 10 m walk time (see Table 6).

Contrary to hypotheses, none of the variables entered in the regression model predicting total MET minutes were associated with this self-report measure of physical activity.

## 4. Discussion

The current study found some support for the hypothesis that youth at increased risk for chronic pain, due to family history, experience different neighborhood characteristics. Low-risk adolescents were more likely than their high-risk counterparts to live in walkable neighborhoods, have nearby parks, and have a larger proportion of college graduates in their neighborhood. This supports the idea that having a parent with a chronic pain condition likely represents a complex set of risk factors. A recently published model of the intergenerational transmission of pain proposes genetic, neurobiological, psychological, behavioral, stress, and health mechanisms of risk for chronic pain in the offspring of youth [41]. The results of the current study provide some initial support for neighborhood characteristics differing in high-risk youth, suggesting that factors outside of the immediate family environment may also play a role, or further contribute to children’s stress or poor health.

Additionally, these neighborhood level characteristics, specifically proximity to parks and walkability, contributed to pain frequency, pain-related activity limitations and a lab-based test of physical function, above the effect of study group status and BMI percentile. This suggests that the built environment may contribute additional unique variance to these outcomes. This could be related to increased or decreased levels of physical activity based on available outlets. Higher neighborhood walkability was associated with elevated performance on the timed 10 m walk test, such that adolescents living in more walkable neighborhoods walked at a quicker pace in the laboratory. This was above and beyond the contribution of study group and BMI. Previous research on neighborhood walkability has demonstrated that increased walkability of the neighborhood is associated with individual’s likelihood to walk for daily transportation, and reduces risk for individuals being overweight or obese [42]. Additionally, moving to a neighborhood with a 10-point increase in walkability leads to increased walking and decreased BMI [43], suggesting that these neighborhood characteristics have the potential to change important health behaviors and health indicators. Regular exercise or lack thereof may contribute directly to pain symptoms, perceived activity limitations, and physical function. For instance, recent research has demonstrated that aerobic exercise prior to surgery is associated with reduced pain after surgery [44], suggesting that regular exercise may be protective against the development of chronic pain. Among adolescents, the regular exercise that is associated with the built environment might also be associated with reduced pain. In the current study, closer proximity to parks was associated with lower pain frequency and lower pain-related activity limitations, indicating that there may be a relationship between the availability of parks or greenspace and the perceived limitations that an adolescent experiences due to pain. In addition to the exercise opportunities provided by parks and green spaces, access to green space has been associated with reduced psychological distress [45]. Psychological distress (e.g., depression, anxiety) plays an important role in pain persistence and pain-related disability among children and adolescents [46,47,48], and proximity to parks may make additional contributions to psychological function.

Negative attitudes about physical activity were associated with both physical functioning tests and experience of pain in the current study. Participants with more negative attitudes about physical activity scored lower on the physical functioning tests, and higher on perceived activity limitations (CALI-21). Negative attitudes about physical activity were correlated with longer timed 10 m walks, and poorer performance in the sit-to-stand test. Inversely, positivity about physical activity is correlated with a shorter timed 10 m walk. These results are consistent with past research that activity level is predicted by attitudes toward activity [13]. It appears that attitudes about physical activity influence the pace or eagerness of engaging in physical activity, even in a laboratory setting. The correlation between CALI-21 scores and negative attitudes about physical activity suggests that negative attitudes about physical activity may lead adolescents to perceive themselves as more limited in their ability to do activities because of pain. Participants with negative attitudes about physical activity were more likely to have more frequent and more intense pain. An individual that experiences frequent and intense pain is likely to have negative feelings about physical activity in the event that such activity exacerbates the pain. Contrary to prior research with youth with chronic pain [15], BMI was not a significant predictor of perceived activity limitations; however, higher BMI was associated with decreased ability to perform activity tasks. The discrepancy with prior literature could be explained by the difference in sample, with previous literature focusing primarily on children with chronic pain conditions and the current study examining a non-clinical sample of children.

Overall, group membership was strongly associated with the majority of the dependent variables that were analyzed in this study. This finding provides further support for past literature indicating that there is a strong relationship between parental chronic pain and chronic pain in adolescent offspring [20]. Being a member of the low-risk group alone was associated with decreased pain intensity, frequency, and perceived activity limitations in this sample. This study adds to the literature in terms of our understanding of the impact of parental chronic pain on offspring physical functioning, as the high-risk group in the current sample demonstrated poorer physical function on performance tests.

Contrary to hypotheses, neither family nor neighborhood-level SES variables were significantly associated with pain or activity limitations after accounting for study group. This finding may indicate that having a parent with chronic pain represents a significant risk factor in itself, and this risk may account for or include SES factors. The study also did not find support for parental chronic pain or any neighborhood characteristics predicting youth self-report of physical activity. Assessment of physical activity via self-report may be problematic in this age group, or this measure may be systematically biased in the high-risk group. Future studies might include objective measures of physical activity such as actigraphy [19,49].

The current study adds to our understanding of the potential role that the neighborhood and built environment might play in increasing or decreasing risk for pain, activity limitations, and physical function. Overall, the models tested in the current study accounted for approximately 18% to 40% of the variance in outcomes, with the built environment variables being significant in a number of models. Findings of this study support the upstream public health concept that built environments can play a pivotal role in the health of its residents [50]. Additionally, this study provides information about how specific environmental characteristics are associated with functioning, which can better inform caregivers and medical providers in facilitating achievement of optimal success in pain-reduction and pain-management [50]. Findings from this study suggest that increasing accessibility to parks and greenspaces and shifting attitudes surrounding physical activity may result in decreased pain and reduced psychological distress, which should be examined in future longitudinal studies. The study had several strengths, including the use of individual address-based data about neighborhood walkability and proximity to parks (vs. relying on participant self-report of neighborhood features). The use of objective measures of physical function is also a strength.

Future research should focus on further delineating how patients’ built environments around their homes can affect their recovery and ability to strive for optimal health. Additionally, longitudinal research would increase knowledge of temporal relationships and changes over time. It will also be important to test more complex models of associations among predictors and outcomes in longitudinal data sets, including mediation models. For instance, it is possible that the built environment influences attitudes, which in turn impact health behaviors and pain outcomes. Future studies might examine these kinds of complex models over time.

There are several limitations that should be kept in mind when interpreting these results. First and foremost, the data is cross-sectional, and as such no inference about directionality or causality of the observed associations can be made. Studies examining this topic have not been longitudinal, which precludes research in this area from commenting on pain development. Additionally, the relatively small sample size likely limited the statistical power and ability to detect smaller effect sizes.

## 5. Conclusions

The current study adds to our understanding of the role that neighborhood features may play in adolescent pain and related functioning. Neighborhood characteristics appear to influence increasing or decreasing risk for pain, activity limitations, and physical function. Adolescents at a lower risk for developing chronic pain were more likely to live in walkable neighborhoods, have nearby parks, and have a larger proportion of college graduates in their neighborhood. These neighborhood level characteristics relate to pain frequency, pain-related activity limitations, and a laboratory-based test of physical function, above the effect of study group status and BMI percentile, suggesting that the built environment may contribute additional unique variance to these outcomes. Negative attitudes about physical activity also made important contributions to pain characteristics and other outcomes. Future studies of risk for chronic pain should consider including assessment of neighborhood characteristics.

## Figures and Tables

**Table 1:** Neighborhood characteristics: Comparison between high-risk and low-risk adolescents.

	High-Risk		Low-Risk						
	Mean	Standard Deviation	Mean	Standard Deviation	Mean Difference	95% Confidence Interval	d	*t*	*p* Value
**Walk Score**	34.27	23.4	49.61	24.54	15.34	7.13, 23.55	0.64	3.694 **	<0.001
**Park Score**	1.49	0.77	1.76	0.52	0.27	0.50, 0.49	0.41	2.430 *	0.016
**Percent Below Poverty Level**	15.86	7.27	15.94	7.78	0.08	−2.49, 2.65	0.01	0.063	0.950
**Median Household Income ($)**	73,446.74	21,643.12	73,103.51	18,152.98	−343.23	−7092.41, 6405.95	0.02	−0.101	0.920
**Percent College Graduates**	21.74	9.01	25.24	7.31	3.50	0.74, 6.26	0.43	2.511 *	0.013

* *p* <0.05, ** *p* < 0.001.

**Table 2:** Multiple regression model of adolescent usual pain intensity.

Variable	Step 1			Step 2			Step 3		
	Unstandardized		Standardized	Unstandardized		Standardized	Unstandardized		Standardized
	B	*SE* B	β	B	*SE* B	β	B	*SE* B	β
**Group ^a^**	0.888	0.301	0.259 *	1.0006	0.319	0.293 *	0.818	0.309	0.239 *
**Body Mass Index (BMI)**	0.006	0.005	0.104	0.007	0.005	0.112	0.005	0.005	0.089
**Park Score**				−0.252	0.247	−0.097	−0.098	0.240	−0.038
**Walk Score**				0.011	0.007	0.160	0.010	0.006	0.154
**Positive PA Attitudes**							0.007	0.037	0.016
**Negative PA Attitudes**							0.097	0.026	0.322 *
***R^2^***		0.084			0.106			0.202	
**F change for *R^2^***		5.498 **			1.439			6.997 ***	
**Total Model F**								4.89 ***	

* *p* < 0.05, ** *p* < 0.01, *** *p* < 0.001; ^a^ Coded as 0 = low risk, 1 = high risk; PA: physical activity.

**Table 3:** Multiple regression model of child usual pain frequency.

Variable	Step 1			Step 2			Step 3		
	Unstandardized		Standardized	Unstandardized		Standardized	Unstandardized		Standardized
	B	*SE* B	β	B	*SE* B	β	B	*SE* B	β
**Group ^a^**	0.759	0.255	0.260 *	0.662	0.264	0.226 *	0.475	0.244	0.162 *
**BMI**	0.007	0.004	0.145	0.008	0.004	0.158	0.006	0.004	0.128
**Park Score**				−0.599	0.204	−0.269 *	−0.467	0.190	−0.210 *
**Walk Score**				0.003	0.005	0.059	0.002	0.005	0.038
**Positive PA Attitudes**							0.049	0.029	0.130
**Negative PA Attitudes**							0.097	0.021	0.379 ***
***R^2^***		0.097			0.159			0.313	
**F change for *R^2^***		6.444 **			4.380 *			12.937 ***	
**Total Model F**								8.796 ***	

* *p* < 0.05, ** *p* < 0.01, *** *p* < 0.001; ^a^ Coded as 0 = low risk, 1 = high risk.

**Table 4:** Multiple regression model of child activity limitations.

Variable	Step 1			Step 2			Step 3		
	Unstandardized		Standardized	Unstandardized		Standardized	Unstandardized		Standardized
	B	*SE* B	β	B	*SE* B	β	B	*SE* B	β
**Group ^a^**	7.218	1.583	0.385 ***	7.292	1.630	0.389 ***	5.896	1.462	0.315 ***
**BMI**	0.013	0.027	0.039	0.018	0.026	0.055	0.008	0.023	0.024
**Park Score**				−3.822	1.260	−0.268 *	−2.693	1.138	−0.189 *
**Walk Score**				0.063	0.034	0.171	0.059	0.030	0.162
**Positive PA Attitudes**							0.075	0.176	0.031
**Negative PA Attitudes**							0.719	0.123	0.439 ***
***R^2^***		0.154			0.219			0.399	
**F change for *R^2^***		10.887 ***			4.972 **			17.326 ***	
**Total Model F**								12.831 ***	

* *p* < 0.05, ** *p* < 0.01, *** *p* < 0.001; ^a^ Coded as 0 = low risk, 1 = high risk.

**Table 5:** Multiple regression model of sit-to-stand test.

Variable	Step 1			Step 2			Step 3		
	Unstandardized		Standardized	Unstandardized		Standardized	Unstandardized		Standardized
	B	*SE* B	β	B	*SE* B	β	B	*SE* B	β
**Group ^a^**	−3.933	1.572	−0.209 *	−4.164	1.678	−0.222 *	−3.058	1.623	−0.163
**BMI**	−0.080	0.027	−0.246 *	−0.080	0.027	−0.247 *	−0.078	0.026	−0.240 *
**Park Score**				0.488	1.333	0.034	−0.345	1.287	−0.024
**Walk Score**				−0.024	0.035	−0.064	−0.034	0.033	−0.091
**Positive PA Attitudes**							0.352	0.190	0.150
**Negative PA Attitudes**							−0.452	0.132	−0.284 **
***R^2^***		0.116			0.119			0.215	
**F change for *R^2^***		8.407 **			0.242			7.561 **	
**Total Model F **								5.667 **	

* *p* < 0.05, ** *p* < 0.001; ^a^ Coded as 0 = low-risk, 1 = high-risk.

**Table 6:** Multiple regression of 10-meter walk time.

Variable	Step 1			Step 2			Step 3		
	Unstandardized		Standardized	Unstandardized		Standardized	Unstandardized		Standardized
	B	*SE* B	β	B	*SE* B	β	B	*SE* B	β
**Group ^a^**	0.427	0.162	0.225 *	0.496	0.171	0.261 *	0.412	0.168	0.217 *
**BMI**	0.005	0.003	0.152	0.005	0.003	0.155	0.005	0.003	0.154
**Park Score**				−0.139	0.136	−0.095	−0.074	0.133	−0.050
**Walk Score**				0.007	0.004	0.186	0.008	0.003	0.218 *
**Positive PA Attitudes**							−0.048	0.020	−0.201 *
**Negative PA Attitudes**							0.030	0.014	0.186 *
***R^2^***		0.081			0.110			0.180	
**F change for *R^2^***		5.675 **			2.005			5.317 **	
**Total Model F**								4.539	

* *p* < 0.05, ** *p* < 0.01; ^a^ Coded as 0 = low-risk, 1 = high-risk.

## References

[1] Palermo T.M., Riley C.A., Mitchell B.A. (2008). Daily functioning and quality of life in children with sickle cell disease pain: Relationship with family and neighborhood socioeconomic distress. J. Pain.

[2] Duncan D.T., Sharifi M., Melly S.J., Marshall R., Sequist T.D., Rifas-Shiman S.L., Taveras E.M. (2014). Characteristics of walkable built environments and BMI *z*-scores in children: evidence from a large electronic health record database. Environ. Health Perspect..

[3] Roth-Isigkeit A., Thyen U., Stoven H., Schwarzenberger J., Schmucker P. (2005). Pain among children and adolescents: Restrictions in daily living and triggering factors. Pediatrics.

[4] De Meester F., Van Dyck D., De Bourdeaudhuij I., Deforche B., Sallis J.F., Cardon G. (2012). Active living neighborhoods: Is neighborhood walkability a key element for Belgian adolescents?. BMC Public Health.

[5] Sallis J.F., Floyd M.F., Rodriguez D.A., Saelens B.E. (2012). Role of built environments in physical activity, obesity, and cardiovascular disease. Circulation.

[6] Beard J.R., Blaney S., Cerda M., Frye V., Lovasi G.S., Ompad D., Rundle A., Vlahov D. (2009). Neighborhood characteristics and disability in older adults. J. Gerontol. B Psychol. Sci. Soc. Sci..

[7] Singh G.K., Siahpush M., Kogan M.D. (2010). Neighborhood socioeconomic conditions, built environments, and childhood obesity. Health Aff. (Project Hope).

[8] Andersson H.I., Ejlertsson G., Leden I., Rosenberg C. (1993). Chronic pain in a geographically defined general population: Studies of differences in age, gender, social class, and pain localization. Clin. J. Pain.

[9] Spilsbury J.C., Storfer-Isser A., Kirchner H.L., Nelson L., Rosen C.L., Drotar D., Redline S. (2006). Neighborhood disadvantage as a risk factor for pediatric obstructive sleep apnea. J. Pediatr..

[10] Diez Roux A.V., Merkin S.S., Arnett D., Chambless L., Massing M., Nieto F.J., Sorlie P., Szklo M., Tyroler H.A., Watson R.L. (2001). Neighborhood of residence and incidence of coronary heart disease. N. Engl. J. Med..

[11] Johannes C.B., Le T.K., Zhou X., Johnston J.A., Dworkin R.H. (2010). The prevalence of chronic pain in United States adults: Results of an Internet-based survey. J. Pain.

[12] Fuentes M., Hart-Johnson T., Green C.R. (2007). The association among neighborhood socioeconomic status, race and chronic pain in black and white older adults. J. Natl. Med. Assoc..

[13] Sallis J.F., Prochaska J.J., Taylor W.C. (2000). A review of correlates of physical activity of children and adolescents. Med. Sci. Sports Exerc..

[14] Gordon-Larsen P., Nelson M.C., Page P., Popkin B.M. (2006). Inequality in the built environment underlies key health disparities in physical activity and obesity. Pediatrics.

[15] Wilson A.C., Samuelson B., Palermo T.M. (2010). Obesity in children and adolescents with chronic pain: Associations with pain and activity limitations. Clin. J. Pain.

[16] Hainsworth K.R., Davies W.H., Khan K.A., Weisman S.J. (2009). Co-occurring chronic pain and obesity in children and adolescents: The impact on health-related quality of life. Clin. J. Pain.

[17] Hershey A.D., Powers S.W., Nelson T.D., Kabbouche M.A., Winner P., Yonker M., Linder S.L., Bicknese A., Sowel M.K., McClintock W. (2009). Obesity in the pediatric headache population: A multicenter study. Headache.

[18] Smith S.M., Sumar B., Dixon K.A. (2014). Musculoskeletal pain in overweight and obese children. Int. J. Obes. (Lond.).

[19] Wilson A.C., Palermo T.M. (2012). Physical activity and function in adolescents with chronic pain: A controlled study using actigraphy. J. Pain.

[20] Higgins K.S., Birnie K.A., Chambers C.T., Wilson A.C., Caes L., Clark A.J., Lynch M., Stinson J., Campbell-Yeo M. (2015). Offspring of parents with chronic pain: A systematic review and meta-analysis of pain, health, psychological, and family outcomes. Pain.

[21] Harris P.A., Taylor R., Thielke R., Payne J., Gonzalez N., Conde J.G. (2009). Research electronic data capture (REDCap)—A metadata-driven methodology and workflow process for providing translational research informatics support. J. Biomed. Inform..

[22] Lester N., Lefebvre J.C., Keefe F.J. (1994). Pain in young adults: I. Relationship to gender and family pain history. Clin. J. Pain.

[23] Currie C., Samdal O., Boyce W., Smith R. (2001). Health Behaviour in School-Aged Children: A WHO Cross-National Study (HBSC): Research Protocol for the 2001/2002 Survey.

[24] Stanford E.A., Chambers C.T., Biesanz J.C., Chen E. (2008). The frequency, trajectories and predictors of adolescent recurrent pain: A population-based approach. Pain.

[25] Palermo T.M., Valenzuela D., Stork P.P. (2004). A randomized trial of electronic versus paper pain diaries in children: Impact on compliance, accuracy, and acceptability. Pain.

[26] Peterson C.C., Palermo T.M. (2004). Parental reinforcement of recurrent pain: The moderating impact of child depression and anxiety on functional disability. J. Pediatr. Psychol..

[27] Palermo T.M., Lewandowski A.S., Long A.C., Burant C.J. (2008). Validation of a self-report questionnaire version of the Child Activity Limitations Interview (CALI): The CALI-21. Pain.

[28] Palermo T.M., Witherspoon D., Valenzuela D., Drotar D. (2004). Development and validation of the Child Activity Limitations Interview: A measure of pain-related functional impairment in school-age children and adolescents. Pain.

[29] Arvidsson D., Slinde F., Hulthen L. (2005). Physical activity questionnaire for adolescents validated against doubly labelled water. Eur. J. Clin. Nutr..

[30] Craig C.L., Marshall A.L., Sjostrom M., Bauman A.E., Booth M.L., Ainsworth B.E., Pratt M., Ekelund U., Yngve A., Sallis J.F. (2003). International physical activity questionnaire: 12-country reliability and validity. Med. Sci. Sports Exerc..

[31] Nelson T.D., Benson E.R., Jensen C.D. (2010). Negative attitudes toward physical activity: Measurement and role in predicting physical activity levels among preadolescents. J. Pediatr. Psychol..

[32] Control C.F.D. BMI Calculator for Child and teen: English Version. http://apps.nccd.cdc.gove/dnpabmi/calculator.aspx.

[33] Radtke T., Puhan M.A., Hebestreit H., Kriemler S. (2016). The 1-min sit-to-stand test—A simple functional capacity test in cystic fibrosis?. J. Cyst. Fibros..

[34] Kumban W., Amatachaya S., Emasithi A., Siritaratiwat W. (2013). Five-times-sit-to-stand test in children with cerebral palsy: Reliability and concurrent validity. NeuroRehabilitation.

[35] Jones C.J., Rikli R.E., Beam W.C. (1999). A 30-s chair-stand test as a measure of lower body strength in community-residing older adults. Res. Q. Exerc. Sport.

[36] Lythgo N., Wilson C., Galea M. (2011). Basic gait and symmetry measures for primary school-aged children and young adults. II: Walking at slow, free and fast speed. Gait Posture.

[37] Thompson P., Beath T., Bell J., Jacobson G., Phair T., Salbach N.M., Wright F.V. (2008). Test-retest reliability of the 10-metre fast walk test and 6-minute walk test in ambulatory school-aged children with cerebral palsy. Dev. Med. Child. Neurol..

[38] Graham J.E., Ostir G.V., Fisher S.R., Ottenbacher K.J. (2008). Assessing walking speed in clinical research: A systematic review. J. Eval. Clin. Pract..

[39] Tuckel P., Milczarski W. (2015). Walk Score (TM), Perceived Neighborhood Walkability, and walking in the US. Am. J. Health Behav..

[40] Duncan D.T., Aldstadt J., Whalen J., Melly S.J., Gortmaker S.L. (2011). Validation of Walk Score^®^ for estimating neighborhood walkability: An analysis of four US metropolitan areas. Int. J. Environ. Res. Public Health.

[41] Stone A.L., Wilson A.C. (2016). Transmission of risk from parents with chronic pain to offspring: An integrative conceptual model. Pain.

[42] Smith K.R., Brown B.B., Yamada I., Kowaleski-Jones L., Zick C.D., Fan J.X. (2008). Walkability and body mass index. Am. J. Prev. Med..

[43] Hirsch J.A., Diez Roux A.V., Moore K.A., Evenson K.R., Rodriguez D.A. (2014). Change in walking and body mass index following residential relocation: The multi-ethnic study of atherosclerosis. Am. J. Public Health.

[44] Vaegter H.B., Handberg G., Emmeluth C., Graven-Nielsen T. (2016). Preoperative hypoalgesia after cold pressor test and aerobic exercise is associated with pain relief six months after total knee replacement. Clin. J. Pain.

[45] Pope D., Tisdall R., Middleton J., Verma A., van Ameijden E., Birt C., Macherianakis A., Bruce N.G. (2015). Quality of and access to green space in relation to psychological distress: Results from a population-based cross-sectional study as part of the EURO-URHIS 2 project. Eur. J. Public Health.

[46] Tran S.T., Jastrowski Mano K.E., Hainsworth K.R., Medrano G.R., Anderson Khan K., Weisman S.J., Davies W.H. (2015). Distinct influences of anxiety and pain catastrophizing on functional outcomes in children and adolescents with chronic pain. J. Pediatr. Psychol..

[47] Huguet A., Tougas M.E., Hayden J., McGrath P.J., Chambers C.T., Stinson J.N., Wozney L. (2016). Systematic review of childhood and adolescent risk and prognostic factors for recurrent headaches. J. Pain.

[48] Kashikar-Zuck S., Goldschneider K.R., Powers S.W., Vaught M.H., Hershey A.D. (2001). Depression and functional disability in chronic pediatric pain. Clin. J. Pain.

[49] Kashikar-Zuck S., Flowers S.R., Verkamp E., Ting T.V., Lynch-Jordan A.M., Graham T.B., Passo M., Schikler K.N., Hashkes P.J., Spalding S. (2010). Actigraphy-based physical activity monitoring in adolescents with juvenile primary fibromyalgia syndrome. J. Pain.

[50] Frank L.D., Engelke P.O. (2001). The built environment and human activity patterns: Exploring the impacts of urban form on public health. J. Plan. Lit..

